# Eutrophication induces shifts in the trophic position of invertebrates in aquatic food webs

**DOI:** 10.1002/ecy.3275

**Published:** 2021-02-13

**Authors:** Gea H. van der Lee, J. Arie Vonk, Ralf C. M. Verdonschot, Michiel H. S. Kraak, Piet F. M. Verdonschot, Jef Huisman

**Affiliations:** ^1^ Department of Freshwater and Marine Ecology Institute for Biodiversity and Ecosystem Dynamics University of Amsterdam P.O. Box 94240 Amsterdam 1090 GE The Netherlands; ^2^ Wageningen Environmental Research Wageningen UR P.O. Box 47 Wageningen 6700 AA The Netherlands

**Keywords:** aquatic consumers, ecological stoichiometry, food web structure, nutrients, omnivores, primary producers, stable isotopes, trophic position

## Abstract

Changes in the ecological stoichiometry of primary producers may have considerable implications for energy and matter transfer in food webs. We hypothesized that nutrient enrichment shifts the trophic position of omnivores towards herbivory, as the nutritional quality of primary producers increases. This hypothesis was tested by analyzing the ecological stoichiometry and stable isotope signature of primary producers and a wide range of aquatic macroinvertebrates, including primary consumers (herbivores) and secondary consumers (both potential omnivores and strict carnivores), along a eutrophication gradient in an agricultural landscape. Our results showed (1) that carbon : nutrient ratios of primary producers decreased along the eutrophication gradient, while the elemental composition of consumers remained homeostatic, and (2) that the trophic position of several omnivores and the generalist predator *Notonecta* decreased, while the trophic position of most other consumers remained constant. These findings suggest that shifts in the diets of aquatic invertebrates induced by increasing eutrophication may affect species interactions and food web structure in aquatic ecosystems.

## Introduction

Agricultural expansion and intensification are among the most predominant human drivers of global environmental change (Tilman et al. 2001). Excessive application of nitrogen (N) and phosphorus (P) fertilizers in agriculture has resulted in eutrophication of surrounding water bodies, with high abundances of algae and macrophytes in lakes, rivers, and streams (Schindler 1974, Smith et al. 1999, Hilton et al. 2006, Huisman et al. 2018) that are often accompanied by major changes in species composition and ecosystem functioning (Conley et al. 2009, Guignard et al. 2017). Enrichment of surface waters through runoff and leaching of nutrients from agricultural lands is therefore considered to be one of the major global water quality issues (Smith et al. 1999, Smith and Schindler 2009).

In addition to changes in species composition and abundances, an increased nutrient supply often also alters the elemental composition of primary producers (Sterner and Elser 2002, Burson et al. 2016). As described by the rapidly advancing field of ecological stoichiometry (Sterner and Elser 2002), changes in the elemental composition of primary producers may have major implications for the trophic transfer of primary production in food webs (Acharya et al. 2004, Moe et al. 2005, Branco et al. 2018). More specifically, primary producers have a high degree of flexibility in their C:N:P stoichiometry. As a consequence, increased nutrient loading tends to result in lower carbon (C)  : nutrient ratios in their tissues (Sterner and Elser 2002, Garbey et al. 2004, Finlay and Kendall 2007, Persson et al. 2010), accompanied by biochemical changes in the production of carbohydrates, amino acids, nucleic acids, and fatty acids (Vrede et al. 2004, Grosse et al. 2017). Nutrient enrichment therefore often improves the nutritional quality of primary producers as food for potential consumers (Elser et al. 2001).

In contrast to primary producers, most consumer species tend to maintain a relatively constant (homeostatic) elemental composition of their tissues, even if the nutritional quality of their food changes (Sterner and Elser 2002, Frost et al. 2003, Evans‐White et al. 2005). Increased nutrient loading thus decreases the elemental imbalance between primary producers and primary consumers (herbivores), thereby reducing stoichiometric constraints on the metabolism and growth of animals eating plant material. This implies that primary consumers feeding on more nutritious plants or algae can employ energetically less costly mechanisms to meet their nutrient uptake, assimilation and retention (Sterner and Hessen 1994, Frost et al. 2003, Sardans et al. 2012, Schoo et al. 2012, Teurlincx et al. 2017). Most secondary consumers are less likely to encounter these stoichiometric constraints, since their bodies have a comparable elemental composition as their homeostatic food sources, i.e., other animals (Frost et al. 2003).

The lower C :  nutrient ratios of primary producers in response to increased nutrient loading may impact those secondary consumers that have physiological, morphological, and behavioral adaptations that allow them to forage and process both plant and animal material (i.e., potential omnivores; Coll and Guershon 2002). In particular, stoichiometric considerations suggest that such omnivorous consumers may lower their trophic position by decreasing their relative consumption of animal material when simultaneously offered plant material of high nutritional quality. Such dietary shifts from animal to plant material have indeed been found in laboratory choice experiments (e.g., Eubanks and Denno 2000, Janssen et al. 2003, Zhang et al. 2018). Although these laboratory experiments are extremely valuable, field studies are needed to assess if changes in the trophic position of organisms induced by an altered C:N:P stoichiometry of primary producers also occur under natural conditions (Lancaster et al. 2005, Moe et al. 2005).

Trophic interactions of natural populations of consumers in the field can be studied using stable isotope analysis of C and N (Woodward and Hildrew 2002). Stable carbon isotope signatures (δ^13^C) can be used to quantify the contribution of specific food sources to the diet of consumers (Finlay and Kendall 2007, Parnell et al. 2010). Enrichment of the stable nitrogen isotope signature (δ^15^N) provides insight into the trophic position of organisms (Cabana and Rasmussen 1996) and sources and transformations of nitrogen (Diebel and Van der Zanden 2009). Studies on the trophic positions of different taxa suggested that aquatic macroinvertebrates may commonly feed on both plant and animal food (Lancaster et al. 2005) and that the trophic position of these macroinvertebrates may vary substantially across sites (Anderson and Cabana 2007).

Although a few studies assessed how trophic interactions are impacted by increased nutrient loading (Singer and Battin 2007, Bergfur et al. 2009, Baumgartner and Robinson 2017), inherent differences in community composition, especially of more abundant species, between field sites with low and high nutrient loadings have limited the assessment of potential shifts in trophic position of specific taxa. In particular, neglecting variation in taxonomic composition among different locations makes it hard to disentangle the extent to which shifts in the trophic structure of food webs can be attributed to variation in community composition or to variation in, e.g., the nutritional quality of primary producers. To assess potential shifts in trophic positions across a wide range of taxa while controlling for variation in community composition, sampling of the same taxa along a eutrophication gradient may provide an elegant solution.

Our study aims to assess if nutrient enrichment drives changes in the trophic position of macroinvertebrates in aquatic food webs. We hypothesized that with increased nutrient loading omnivores will shift their trophic position from consuming animal material toward increased consumption of primary producers, as the nutritional quality of primary producers increases. To test this hypothesis, we analyzed the ecological stoichiometry (C:N, C:P, N:P) and stable isotope signature (δ^13^C and δ^15^N) of primary producers, primary consumers, and secondary consumers (both potential omnivores and strict carnivores) along a eutrophication gradient in a 675‐m permanent drainage ditch. Due to the ditch’s unique position in the landscape, draining a nature reserve into an agricultural area, this ditch enabled sampling of the same set of taxa along a strong gradient in nutrient loading. First, we investigated the common expectation of ecological stoichiometry (Sterner and Elser 2002) that nutrient enrichment will result in lower C : nutrient ratios of primary producers, while primary and secondary consumers maintain a constant elemental composition. Then, we used the δ^15^N stable isotope signatures to evaluate our hypothesis that secondary consumers with an omnivorous diet will shift their trophic positions along the eutrophication gradient.

## Methods

### Study site

**Fig. 1:**
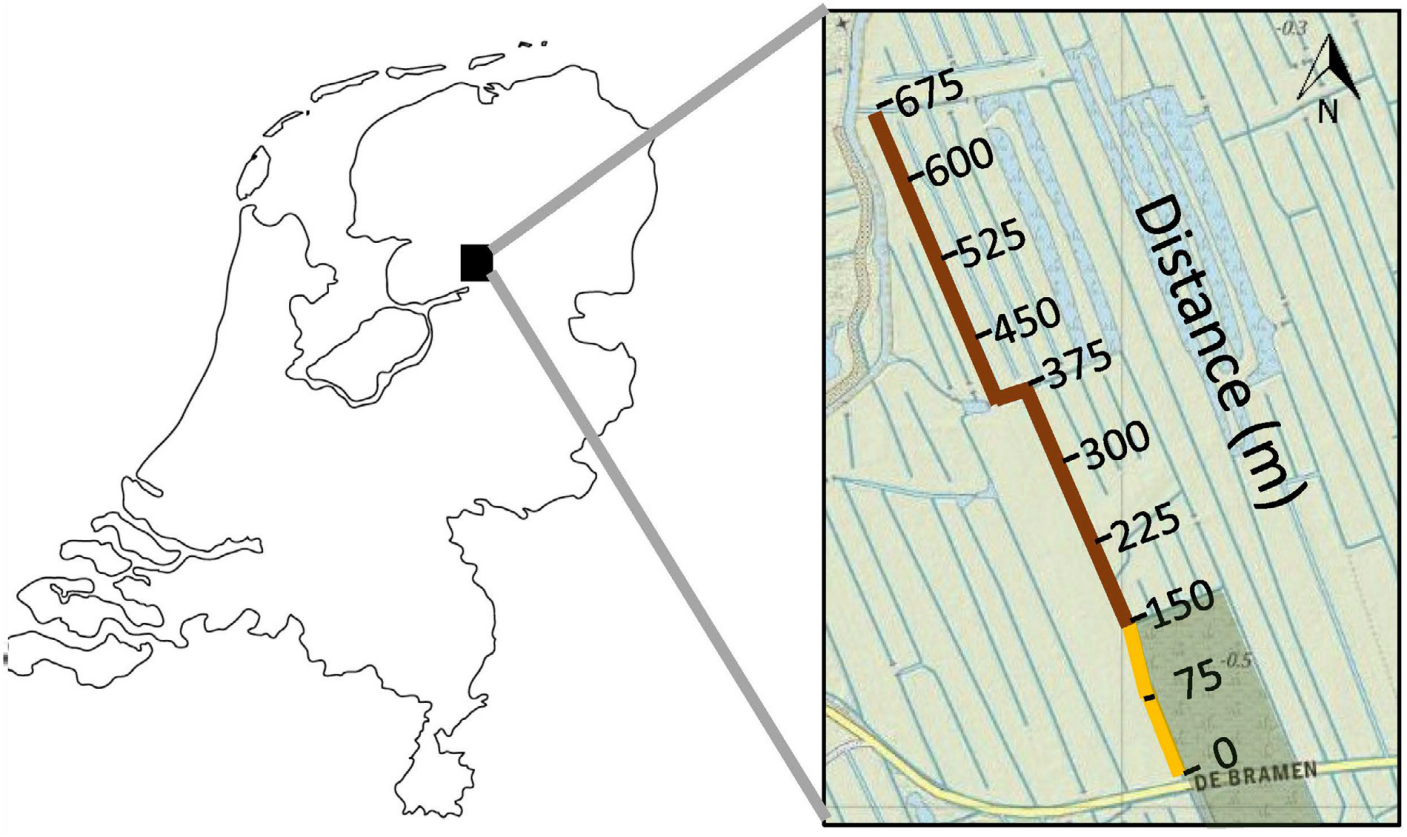
Location of the drainage ditch and division of the ditch into nine 75‐m subsections. The dark green area indicates the nature reserve containing oligo‐ to mesotrophic fen meadows. The yellow line indicates the ditch sections next to the nature reserve and the brown line indicates the ditch sections next to agricultural lands.

The permanent drainage ditch (1–3 m wide, <1 m deep, 0–5 cm/s water flow) studied was located in a peatland area rich in drainage ditches in the north of the Netherlands (Fig. 1). These drainage ditches were originally dug to drain excess water from the surrounding fields. The southern section (52°44'14.7" N, 6°06'49.0" E) of the ditch was positioned adjacent to a nature reserve containing oligo‐ to mesotrophic fen meadows, while at the northern section (52°44'35.0" N, 6°06'33.7" E), land use consisted of intensively farmed agricultural fields grazed by cattle (up to 1.5 large‐sized livestock/ha), fertilized with 10–15 metric tons of stable manure per hectare per year. The change in macrophyte community composition along the length of the ditch indicated the presence of a strong nutrient enrichment gradient (Janse and Van Puijenbroek 1998). It shifted from a species‐rich wetland‐plant community with numerous growth forms and many open‐water areas in the first section of the ditch adjacent to the nature reserve, to dense beds of submerged vegetation and filamentous algae filling the water column in the mid‐section of the ditch, and then to open water with some emergent vegetation and duckweed toward the last section next to the agricultural fields.

### Sampling

The ditch was sampled for elemental and stable isotope analysis in late October 2017 and 2018. The 675 m long ditch was first divided into nine 75‐m subsections. In the middle of each subsection, we collected bottom sediment, suspended matter from the water column, graminoid vegetation from the banks, epiphyton from reed (*Phragmites australis*), four species of macrophytes, and 17 macroinvertebrate taxa. For the sediment, the top 2 cm of the bottom substrate of the ditch was collected in triplicate samples of 50‐mL tubes. For suspended matter, we did not apply a filtration step because the high concentrations of suspended matter in the ditch would rapidly clog the filter. Instead, triplicate samples of 1 L of water were collected in plastic bottles, which were allowed to settle overnight at 4°C before carefully drawing up 100 mL from the bottom of the bottle using a syringe. The remaining water was removed from the subsample by freeze‐drying. In each subsection, plant and animal material was collected as bulk samples without replication. Graminoid vegetation was collected from the banks next to the ditch. Epiphyton (i.e., biofilms attached to submerged plants) was scraped from several new shoots of *Phragmites australis* stems using a blade. Macrophyte taxa were selected based on their occurrence throughout the entire ditch and consisted of submerged *Elodea nuttallii* and *Lemna trisulca*, summer‐floating *Stratiotes aloides*, and emergent *P. australis*. Aboveground parts of all macrophytes were collected by hand and washed with ambient water to remove any attached material and macroinvertebrates.

The consumer community of aquatic macroinvertebrates was collected by sweeping a 0.5‐mm mesh hand net through the submerged vegetation and over the top of the sediment of the ditch. The animals were transported to the laboratory on the same day and were kept one to two nights at 4°C under aerated conditions to allow for gut clearance (Evans‐White et al. 2005). Seventeen invertebrate taxa were selected based on their occurrence throughout the entire 675‐m ditch section. Primary consumers included molluscs (Bivalvia: Sphaeriidae; Gastropoda: *Bithynia* spp., *Lymnaea* sp., *Planorbarius* sp., *Planorbis* spp., *Valvata* spp.), insects (Ephemeroptera: *Cloeon* spp.) and crustaceans (Isopoda: Asellidae; Amphipoda: Crangonyctidae/Gammaridae) that obtain their food solely as collector‐gatherers, shredders, collector‐filterers, or scrapers (see Appendix S1: Table S1 for the assignment of functional feeding groups). Secondary consumers comprised (1) strict carnivores including insects (*Ilyocoris* sp. and *Notonecta* spp.; Odonata: Anisoptera and Zygoptera) and leeches (Hirudinea: *Erpobdella* spp.) that obtain their food solely by predating or piercing animals, and (2) potential omnivores represented by a variety of insects (Coleoptera: *Noterus* spp.; Heteroptera: Corixinae (*Hesperocorixa* spp./*Sigara* spp.) and Trichoptera: Phryganeidae (*Agrypnia* spp./*Phryganea* spp.)) that have the ability to obtain their food both by predating or piercing animals and as collector‐gatherers or shredders. For large taxa, at least two or three individuals were collected and, for small taxa, approximately 15–30 individuals were collected (Evans‐White et al. 2005, Bergfur et al. 2009). Mollusc shells and caddisfly cases were removed, while whole organisms were used for all other taxa. Samples were stored at −20°C.

### Elemental and stable isotope analysis

Prior to analysis all samples were freeze dried. The sediment was dry sieved (2 mm mesh) to remove mollusc shell fragments and dead plants. Thereafter, the samples were ground to fine powder using a ball‐mill for 5 min at 400 rpm for the sediment, an herb grinder for the plants, and a mortar and pestle for the macroinvertebrates. For total C, total N, δ^13^C, and δ^15^N, 5–20 mg freeze‐dried material was weighed to the nearest 0.01 mg in tin capsules and analyzed using a Vario Isotope elemental analyzer (Elementar Analysesysteme GmbH, Langenselbold, Germany) in conjunction with an Bio Vision isotope ratio mass spectrometer (Elementar UK, Manchester, UK). For total P, a 1–20 mg sample was digested using 250 µL HNO_3_ (65%) and 125 µL H_2_O_2_ (30%) in a microwave assisted system (Multiwave 3000, rotor 64MG5, Anton Paar GmbH, Graz, Austria) operated at 350 W for 20 minutes with a 10‐minute ramp and 450 W for 30 minutes with a 5‐minute ramp (Cedergreen et al. 2013), diluted to 4.6 mL and analyzed using an inductively coupled plasma optical emission spectrometer (ICP‐OES, PerkinElmer Optima 8300, Waltham, Massachusetts, USA). Some low mass samples were not analysed for total P. The precision (mean ± SD) of our standards were as follows: δ^13^C, −30.36‰ ± 0.03‰; δ^15^N, 0.69‰ ± 0.10‰; C, 72.07% ± 0.32%; and N, 10.9% ± 0.06% for acetanilide 99% (Sigma‐Aldrich, St. Louis, Missouri, USA) and P, 0.11% ± 0.01% for Granodiorite (Silver Plume, Colorado, USA, GSP‐2).

Isotope ratios were expressed as delta (δ) values, in parts per mil (‰), according to the equation

$$\delta X=\left(\frac{R_{\text{sample}}}{R_{\text{sample}}}‐1\right)\times 1,000$$

where *R*_sample_ is the stable isotope ratio (^13^C/^12^C or ^15^N/^14^N) between the heavy and light isotope in the sample and *R*_standard_ is the stable isotope ratio of the standard reference material (Peedee Belemnite carbonate for δ^13^C; atmospheric N_2_ for δ^15^N). A higher delta value indicates that the sample is more enriched in the heavy isotope (Fry 2006). Additionally, we calculated the trophic position (TP) of each consumer following the simplest model (Post 2002)

$$\text{TP}=\lambda +\frac{\left(\delta^{15}N_{\text{consumer}}‐\delta^{15}N_{\text{base}}\right)}{\Delta_{n}}$$

where λ is the trophic position of the organisms at the baseline, δ^15^N_base_ and δ^15^N_consumer_ are the δ^15^N values of the organisms at the baseline and of the consumer, respectively, and Δ*_n_* is the expected enrichment in δ^15^N per trophic level. We used the mean δ^15^N of the primary producers (λ = 1) to establish the δ^15^N baseline at each ditch section and used Δ*_n_* = 2.55 based on the compilation of three meta‐analyses by Matthews and Mazumder (2008).

### Statistical analysis

To establish the presence of a eutrophication gradient, linear regressions were performed with the total nutrient content (percent C, N, and P), ecological stoichiometry (C:N, C:P, and N:P ratios) and isotope signatures (δ^15^N and δ^13^C) of the ditch sediment, total suspended matter and graminoid vegetation as dependent variables and distance along the ditch (oriented from the nature reserve to the agricultural area) as independent variable. Thereafter, we analyzed how the distance along the ditch, as proxy for the eutrophication gradient, affected the ecological stoichiometry, stable isotope signatures and trophic position of the different trophic groups. For this purpose, we used the following generalized linear mixed model (GLMM) in Wilkinson notation:

$$\text{Response}\;\text{variable}∼\text{distance}\times \text{trophic}\;\text{group}+\left(\text{distance}|\text{trophic}\;\text{group}:\text{taxa}\right).$$

Fixed effects were distance along the ditch (continuous, rescaled from 0 to 1), trophic group (categorical with three levels: primary producers, primary consumer, and secondary consumer), and the interaction between distance and trophic group. To account for taxonomic variation, we included random effects of the slope and intercept among taxa nested within their trophic group. The C:N, C:P, and N:P ratios were log_10_(*x* + 1)‐transformed to improve the normal distribution of the residuals and to reduce heteroscedasticity. The models were fitted using REML and *P* values were derived using the Satterthwaite approximations to degrees of freedom. To estimate the overall fit of the models, we calculated coefficients of determination (pseudo‐*R* ^2^) for the fixed effects only (marginal *R* ^2^) and for the fixed and random effects combined (conditional *R* ^2^) (Nakagawa and Schielzeth 2013, Johnson 2014). A pairwise analysis of the estimated marginal means (EMMs) and estimated slopes of the fitted lines was performed to interpret the final models using a multiplicity adjustment. To allow for interpretation of these results, the log_10_(*x* + 1)‐transformed ecological stoichiometry variables were back‐transformed to the response scale. As we conducted each analysis on six independent variables, Bonferroni correction was applied to correct for multiple hypothesis testing (significance level of 0.05/6 = 0.0083). All analyses were performed in R version 3.6.3. (R Core Team 2019) using the lm function in the stats package to fit the simple linear models, the lmer function in the lme4 package to fit the GLMM (Bates et al. 2015; v. 1.1.21) in combination with the lmerTest package to calculate *P* values (Kuznetsova et al. 2017; v. 3.1.1), the summ function in the jtools package to calculate pseudo‐*R* ^2^ (Long 2020; v. 2.1.0), and the emmeans and lstrends functions in the emmeans package to interpret the final model and back‐transform the results (Lenth 2020; 1.4.7).

## Results

**Fig. 2:**
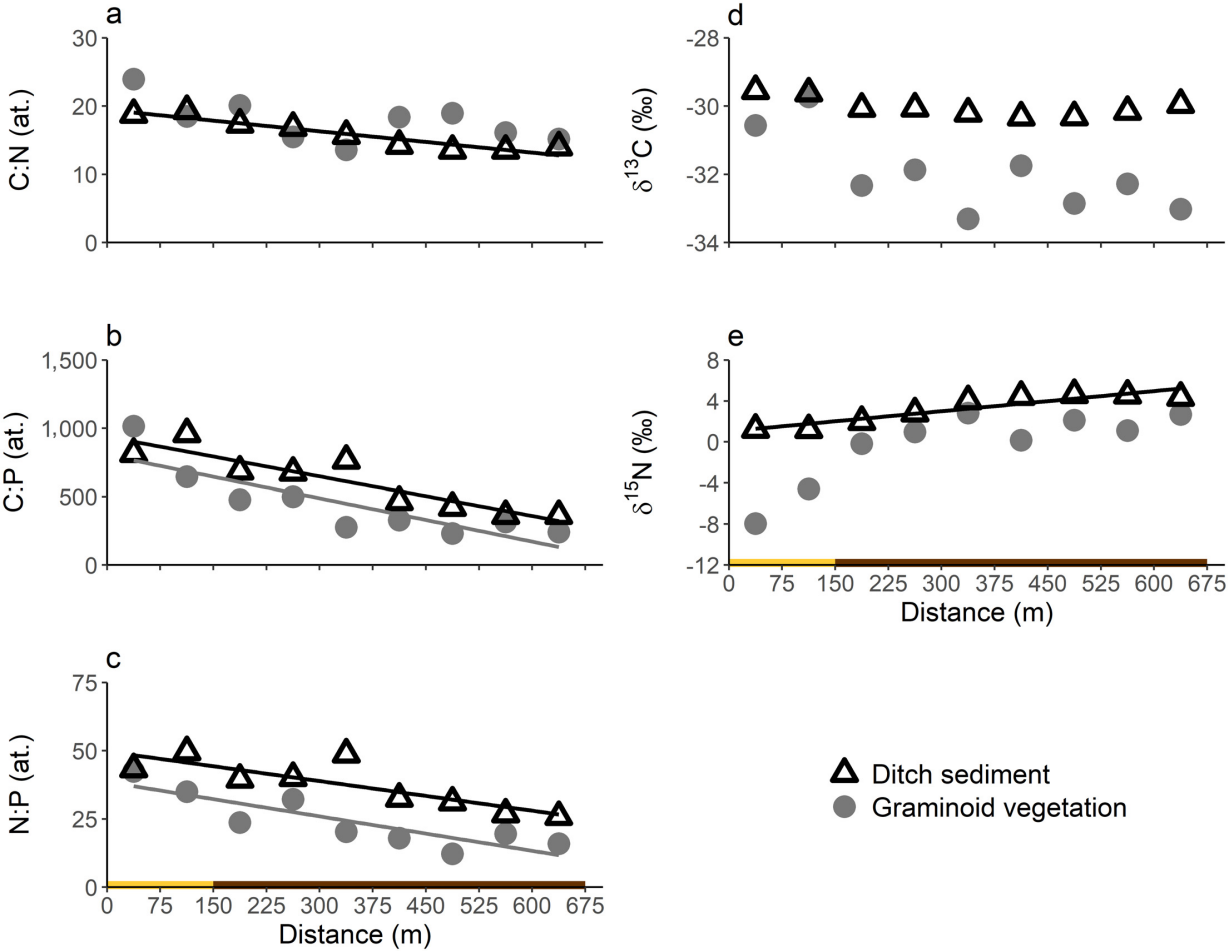
Elemental stoichiometry and stable isotope signature of graminoid vegetation and ditch sediment along the length of the ditch (at. indicates atomic ratio). (a) C:N ratio (graminoids, *R* ^2^ = 0.37, *P* = 0.08; sediment, *R* ^2^ = 0.90, *P* < 0.001), (b) C:P ratio (graminoids, *R* ^2^ = 0.73, *P* = 0.003; sediment, *R* ^2^ = 0.83, *P* = 0.001), (c) N:P ratio (graminoids, *R* ^2^ = 0.75, *P* = 0.003; sediment, *R* ^2^ = 0.70, *P* = 0.005), (d) δ^13^C (graminoids, *R* ^2^ = 0.51, *P* = 0.03; sediment, *R* ^2^ = 0.43, *P* = 0.06), and (e) δ^15^N (graminoids, *R* ^2^ = 0.64, *P* = 0.01; sediment, *R* ^2^ = 0.86, *P* < 0.001). Lines indicate significant linear regressions (using a Bonferroni corrected significance level of *P* < 0.008; *n* = 9 in all graphs). The colored bar below the graphs indicates whether the ditch sections were located adjacent to the nature reserve (yellow bar) or agricultural lands (brown bar).

The existence of a eutrophication gradient was confirmed by a significant increase in P content of the sediments in the ditch (*R* ^2^ = 0.74, *P* = 0.003) and the graminoid vegetation on the banks (*R* ^2^ = 0.80, *P* = 0.001) along the distance of the ditch, from the nature reserve toward the agricultural area (see Appendix S2: Fig. S1). The N content of the ditch sediment showed a marginally significant increase along the ditch (*R* ^2^ = 0.44, *P* = 0.05), while the C content did not change (*R* ^2^ = 0.03, *P* = 0.64). In line with these results, the C:N and C:P ratios of the ditch sediment and C:P ratios of the graminoid vegetation decreased significantly along the ditch (Fig. 2a, b). N:P ratios of the ditch sediment and graminoid vegetation also decreased significantly, indicative of more extensive P than N enrichment in the agricultural area (Fig. 2c).

The δ^13^C signature of the graminoid vegetation and ditch sediment did not change significantly along the ditch (Fig. 2d). In contrast, the δ^15^N signature of the ditch sediment increased significantly along the ditch. The δ^15^N signature of the graminoid vegetation on the banks of the ditch was lower in the nature reserve (first 150 m) than in the agricultural area, but this trend was not significant (Fig. 2e). For suspended matter, no significant changes were detected in elemental stoichiometry and stable isotope signatures (see Appendix S3: Tables S1, S2).

**Fig. 3:**
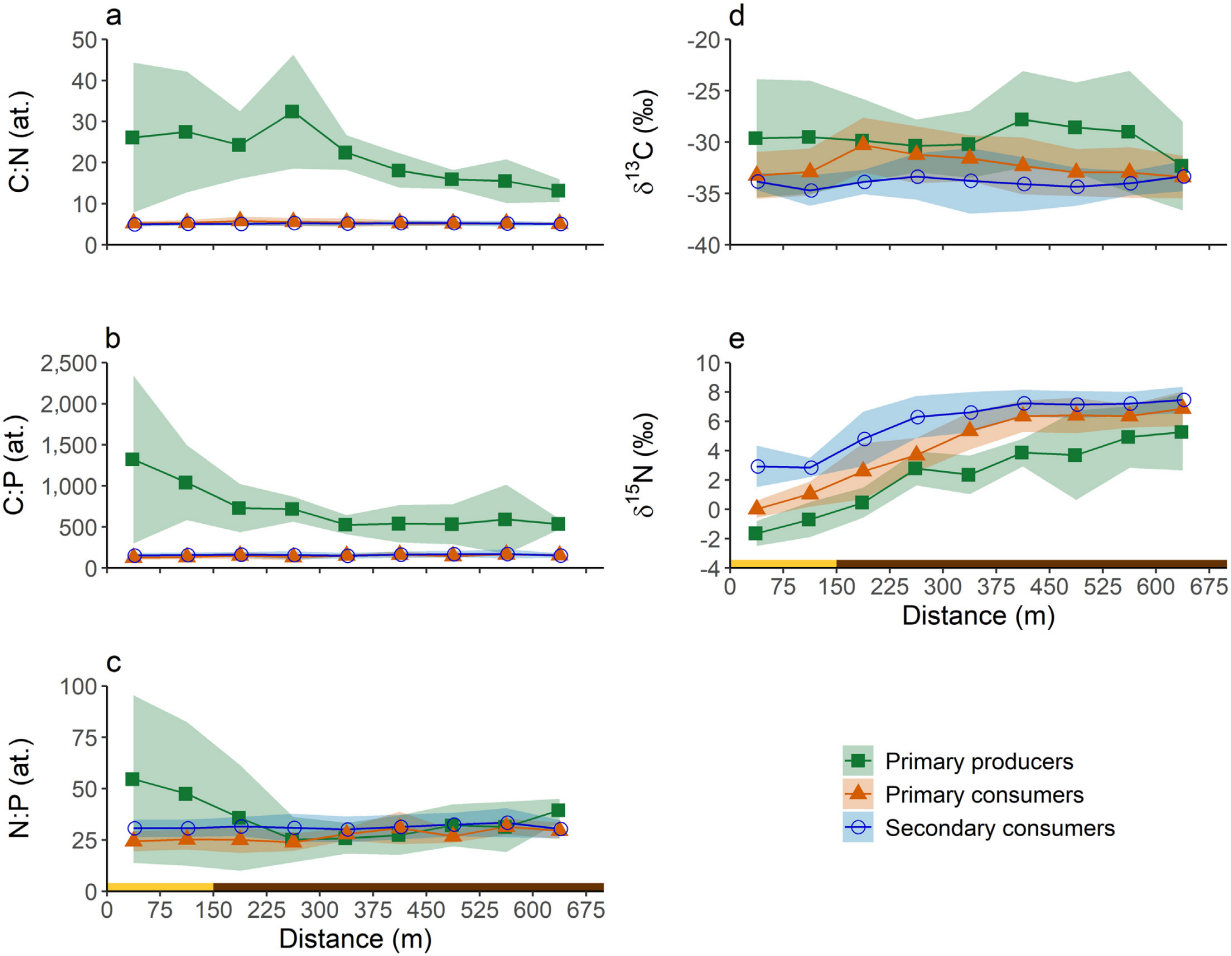
Stoichiometry and stable isotope signature of primary producers, primary consumers, and secondary consumers along the length of the ditch. (a) C:N ratio, (b) C:P ratio, (c) N:P ratio, (d) δ^13^C, and (e) δ^15^N. The mean values (line) ± SD (ribbon) are shown, based on five taxa of primary producers, nine taxa of primary consumers, and eight taxa of secondary consumers. The colored bar below the graphs indicates whether the ditch sections were located adjacent to the nature reserve (yellow bar) or agricultural lands (brown bar). Details of individual taxa are shown in Appendix S3: Figs. S1–S5.

The C:N and C:P ratios of the primary producers were significantly higher than those of the primary and secondary consumers (Fig. 3a, b; Tables 1, 2). Moreover, the C:N and C:P ratios of the primary producers decreased significantly with the distance along the ditch (see the estimated slopes in Table 2). The decline in C:N ratio along the ditch was greatest for the two submerged macrophytes *Elodea nuttallii* and *Lemna trisulca*, while the decline in C:P ratio was most pronounced in the epiphyton (details individual taxa, see Appendix S3: Figs. S1, S2, Table S1). In contrast, the C:N and C:P ratios of the primary and secondary consumers did not decrease significantly with distance along the ditch (as indicated by the 95% confidence intervals of the estimated slopes; Table 2), indicating that these macroinvertebrates remained homeostatic.

**Table 1:** Performance of the GLMM models for ecological stoichiometry (C:N, C:P, and N:P ratios), stable isotope signatures (δ^13^C and δ^15^N) and trophic position, with estimated fixed effects (distance, trophic groups, and their interaction) and random effects (taxonomic composition).

Factor	Estimate	SE	*t*	*P*	σ
C:N ratio (*n* = 180; marginal *R* ^2^ = 0.87; conditional *R* ^2^ = 0.95)					
Fixed effects					
Intercept	1.45	0.05	28.2	2 × 10^−16^*	
Distance	−0.26	0.07	−3.9	9 × 10^−4^*	
Primary consumers	−0.64	0.06	−10.0	6 × 10^−9^*	
Secondary consumers	−0.66	0.07	−10.0	5 × 10^−9^*	
Distance × Primary consumers	0.25	0.08	3.0	0.007*	
Distance × Secondary consumers	0.27	0.09	3.1	0.006*	
Random effects					
Taxa nested in trophic group × Intercept					0.11
Taxa nested in trophic group × Distance					0.14
Residual					0.05
C:P ratio (*n* = 143; marginal *R* ^2^ = 0.82; conditional *R* ^2^ = 0.93)					
Fixed effects					
Intercept	2.99	0.06	46.3	2 × 10^−16^*	
Distance	−0.42	0.12	−3.6	0.002*	
Primary consumers	−0.88	0.08	−10.9	2 × 10^−9^*	
Secondary consumers	−0.80	0.08	−9.5	2 × 10^−8^*	
Distance × Primary consumers	0.53	0.15	3.7	0.002*	
Distance × Secondary consumers	0.46	0.15	3.1	0.007*	
Random effects					
Taxa nested in trophic group × Intercept					0.13
Taxa nested in trophic group × Distance					0.23
Residual					0.08
N:P ratio (*n* = 142; marginal *R* ^2^ = 0.12; conditional *R* ^2^ = 0.60)					
Fixed effects					
Intercept	1.57	0.06	26.2	6 × 10^−16^*	
Distance	−0.17	0.08	−2.2	0.04	
Primary consumers	−0.18	0.08	−2.4	0.03	
Secondary consumers	−0.07	0.08	−0.9	0.4	
Distance × Primary consumers	0.27	0.10	2.8	0.01	
Distance × Secondary consumers	0.19	0.10	1.9	0.07	
Random effects					
Taxa nested in trophic group × Intercept					0.12
Taxa nested in trophic group × Distance					0.13
Residual					0.09
δ^13^C (*n* = 181; marginal *R* ^2^ = 0.22; conditional *R* ^2^ = 0.77)					
Fixed effects					
Intercept	−29.75	1.09	−27.3	2 × 10^−16^*	
Distance	0.59	0.87	0.7	0.5	
Primary consumers	−2.42	1.37	−1.8	0.09	
Secondary consumers	−4.07	1.40	−2.9	0.009	
Distance × Primary consumers	−0.94	1.09	−0.9	0.4	
Distance × Secondary consumers	−0.58	1.12	−0.5	0.6	
Random effects					
Taxa nested in trophic group × Intercept					2.21
Taxa nested in trophic group × Distance					0.65
Residual					1.66
δ^15^N (*n* = 181; marginal *R* ^2^ = 0.75; conditional *R* ^2^ = 0.83)					
Fixed effects					
Intercept	−1.20	0.50	−2.4	0.03	
Distance	7.19	0.70	10.2	6 × 10^−9^*	
Primary consumers	1.99	0.63	3.2	0.006*	
Secondary consumers	4.60	0.65	7.1	3 × 10^−6^*	
Distance × Primary consumers	−0.02	0.88	0.0	1.0	
Distance × Secondary consumers	−2.20	0.90	−2.4	0.03	
Random effects					
Taxa nested in trophic group × Intercept					0.85
Taxa nested in trophic group × Distance					0.89
Residual					1.17
Trophic position (*n* = 138; marginal *R* ^2^ = 0.30; conditional *R* ^2^ = 0.64)					
Fixed effects					
Intercept	1.78	0.16	11.1	2 × 10^−8^*	
Distance	0.04	0.14	0.2	0.8	
Secondary consumers	1.00	0.24	4.1	0.001*	
Distance × Secondary consumers	−0.80	0.21	−3.7	5 × 10^−4^*	
Random effects					
Taxa nested in trophic group × Intercept					0.42
Distance × Secondary consumers					0.15
Residual					0.37

Notes

Reported are the number of observations (*n*), the overall model performance for the fixed effects only (marginal *R* ^2^) and for the fixed and random effects combined (conditional *R* ^2^). For the fixed effects, the regression coefficients (Estimate) with their standard errors (SE), *t* values, and *P* values estimated using the Satterthwaite approximations are presented. For the trophic groups, the primary producers are used as a reference. The fixed effects for the primary consumers and secondary consumers are expressed with respect to this reference level. For the random effects, the standard deviation (σ) is presented. Significant fixed effects are indicated with an asterisk (Bonferroni corrected, *P* < 0.0083). Note that C:N, C:P, and N:P ratios were log_10_(*x* + 1)‐transformed. For details of the individual taxa, see Appendix S3: Table S1, S2.

**Table 2:** Estimated marginal means and estimated slopes along the distance of the ditch obtained by the GLMM models for ecological stoichiometry (C:N, C:P, and N:P ratios), stable isotope signatures (δ^13^C and δ^15^N), and trophic position.

Parameter and trophic group	Estimated marginal mean	Estimated slope
C:N ratio		
Primary producers	19.9^a^ [16.7, 23.2]	−12.7^a^ [−22.4, −3.1]
Primary consumers	5.3^b^ [4.6, 6.1]	−0.2^b^ [−2.1, 1.7]
Secondary consumers	5.2^b^ [4.4, 6.0]	0.0^b^ [−2.0, 2.1]
C:P ratio		
Primary producers	630^a^ [498, 762]	−611^a^ [−1079, −143]
Primary consumers	143^b^ [120, 166]	38^b^ [−38, 113]
Secondary consumers	159^b^ [132, 187]	13^b^ [−78, 104]
N:P ratio		
Primary producers	30.5^a^ [22.9, 38.1]	−12.1^a^ [−27.7, 3.5]
Primary consumers	26.5^a^ [21.4, 31.5]	6.7^a^ [−2.8, 16.2]
Secondary consumers	31.3^a^ [24.7, 37.8]	1.6^a^ [−10.0, 13.2]
δ^13^C		
Primary producers	−29.5^a^ [−32.5, −26.4]	0.6^a^ [−1.7, 2.9]
Primary consumers	−32.4^a^ [−34.6, −30.1]	−0.3^a^ [−2.1, 1.4]
Secondary consumers	−33.8^a^ [−36.2, −31.4]	0.0^a^ [−1.8, 1.9]
δ^15^N		
Primary producers	2.3^a^ [1.3, 3.4]	7.2^a^ [5.3, 9.0]
Primary consumers	4.3^b^ [3.5, 5.1]	7.2^a^ [5.8, 8.5]
Secondary consumers	5.9^c^ [5.0, 6.7]	5.0^a^ [3.5, 6.5]
Trophic position		
Primary producers	na	na
Primary consumers	1.8^a^ [1.5, 2.1]	0.0^a^ [−0.3, 0.4]
Secondary consumers	2.4^b^ [2.1, 2.7]	−0.8^b^ [−1.1, −0.4]

Notes

Different superscript letters indicate significant differences between the trophic groups (Bonferroni correct, *P* < 0.0083). The 95% confidence intervals are given in square brackets. na, not applicable.

The GLMM models for the C:N and C:P ratios fitted well (marginal *R* ^2^> 0.80), while the model for the N:P ratios performed poorly (marginal *R* ^2^ = 0.12) and none of the fixed effects was significant (Table 1). By including random effects, the model performance improved considerably (conditional *R* ^2^ = 0.60), suggesting that there were differences in N:P ratios between taxa, but that these differences were not related to the assigned trophic groups included in the model (Table 1; Fig. 3c; see also Appendix S3: Fig. S3).

The δ^13^C signature of the primary producers and consumers did not change significantly along the distance of the ditch, and did not differ between the trophic groups (Fig. 3d; Tables 1, 2). Explanatory power of the GLMM model improved when random effects were included (marginal *R* ^2^ = 0.22, whereas conditional *R* ^2^ = 0.77; Table 1); indicating that the δ^13^C signature differed between taxa within the same trophic group (see also Appendix S3: Fig. S4). However, the contribution of specific primary producer taxa to the diet of the consumers could not be quantified, as the δ^13^C signatures of most primary producer taxa, with the exception of *Stratiotes aloides,* were very similar to the δ^13^C signatures of the consumers (see Appendix S3: Table S2). In contrast, the δ^15^N signature differed significantly between primary producers, primary consumers and secondary consumers and increased significantly along the ditch gradient (Fig. 3e; Tables 1, 2).

**Fig. 4:**
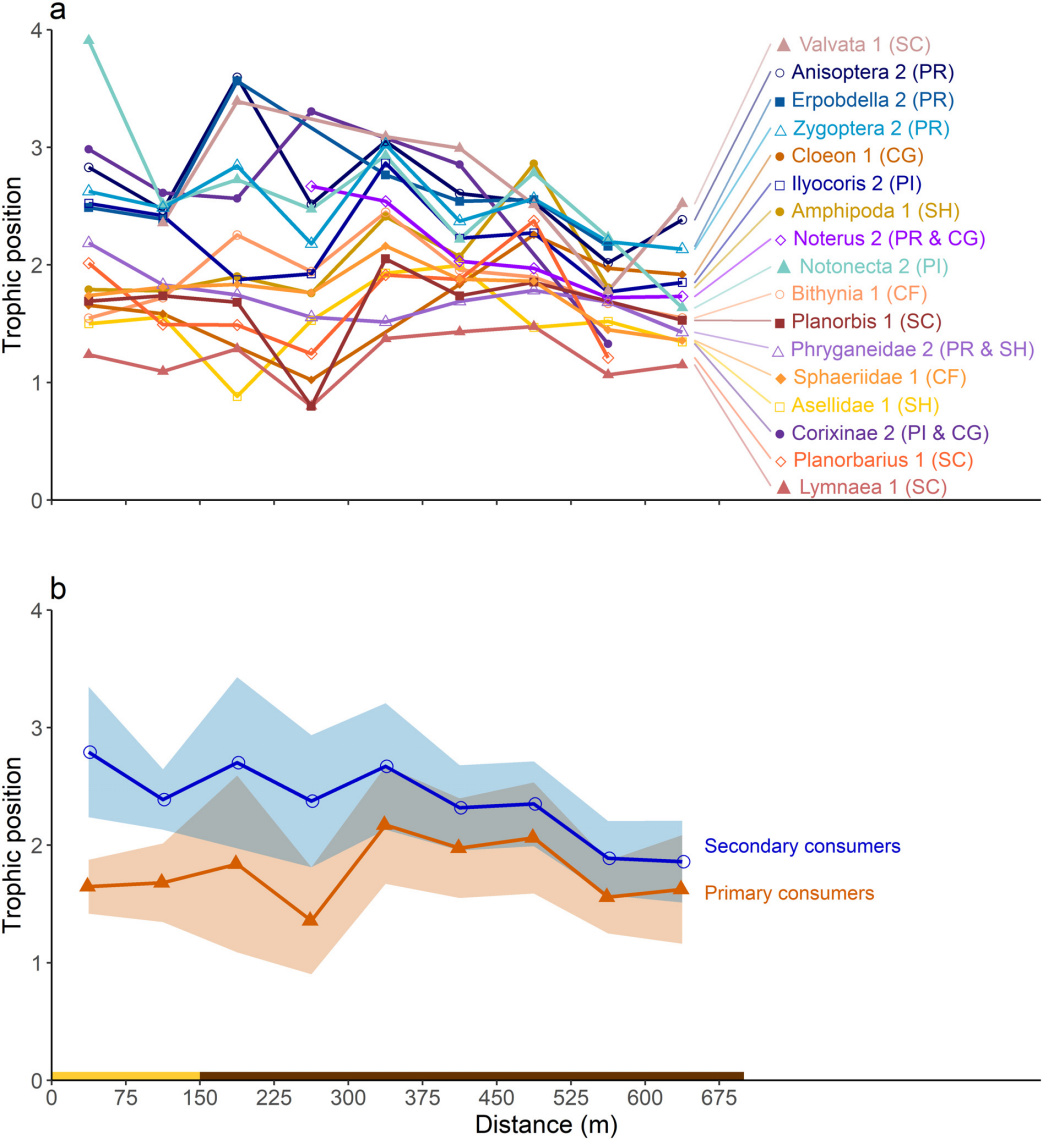
Trophic position of consumers along the length of the ditch. (a) Trophic positions of individual taxa pre‐assigned as primary consumers (1) or secondary consumers (2). (b) The mean trophic position (line) ± SD (ribbon) of the primary consumers (*n* = 9 taxa) and secondary consumers (*n* = 8 taxa). The colored bar below the graphs indicates whether the ditch sections were located next to the nature reserve (yellow bar) or agricultural lands (brown bar). The abbreviations in brackets stand for functional feeding groups of macroinvertebrates: CF, collector‐filterer; CG, collector‐gatherer; SC, scraper; SH, shredder; PR, predator; and PI, piercer (carnivorous).

Trophic positions of the consumer taxa were calculated by comparing their δ^15^N signature against the mean δ^15^N signature of the primary producers. The trophic position of the primary producers was set at 1, and in theory the trophic position of primary consumers should be 2 and that of secondary consumers should be 3. In line with expectation, the trophic positions of taxa pre‐assigned as secondary consumers were generally higher than those of taxa pre‐assigned as primary consumers (Fig. 4a). The mean trophic position of the primary consumers was 1.8, and the estimated slope of 0.0 shows that the trophic position of primary consumers did not change significantly along the distance of the ditch (Fig. 4b; Tables 1, 2). The mean trophic position of the secondary consumers was 2.4, and decreased significantly from the nature reserve to the agricultural fields (Fig. 4b; Tables 1, 2). The model fit improved when random effects were included (marginal *R* ^2^ = 0.30, whereas conditional *R* ^2^ = 0.64; Table 1), indicative of differences between taxa within the trophic groups. Linear regressions of individual taxa revealed that trophic position did not vary along the ditch for any of the primary consumer taxa, but decreased significantly with distance along the ditch for the potential omnivore *Noterus* spp. (*P* = 0.003) and was marginally significant (*P* < 0.05) for the potential omnivore Phryganeidae and the strict carnivore *Notonecta* spp. (Fig. 4a; see Appendix S3: Table S2 for statistical details).

## Discussion

Our results show that the C:N and C:P ratios of primary producers decreased along the eutrophication gradient, while the elemental composition of primary and secondary consumers remained homeostatic. This pattern, which is a common finding of many studies in the field of ecological stoichiometry (e.g., Sterner and Elser 2002), was accompanied by a major downward shift in the trophic position of secondary consumers along the eutrophication gradient, while the trophic position of primary consumers remained constant. The decrease in trophic position of the secondary consumers was associated with taxa that were identified as potential omnivores (i.e., *Noterus* spp. and Phryganeidae) and with a carnivore (*Notonecta* spp.). These results indicate that the omnivorous taxa have adjusted their diet from consumption of primary consumers to increased consumption of primary producers. Studies from terrestrial systems have shown that feeding on both plant and animal material is widespread among macroinvertebrates (see review by Coll and Guershon 2002), and there is increasing evidence that omnivory also occurs among aquatic macroinvertebrates (France 1997, Lancaster et al. 2005, Figueroa et al. 2019). Our results thus provide field support for the idea that macroinvertebrates may change their diet depending on the resources available along productivity gradients (Wootton 2017), which may cause changes in their trophic positions and hence in food web structure.

One of the reasons why omnivores may shift their trophic position downward with increased nutrient loading is that the C : nutrient ratio of primary producers decreases and becomes more comparable to the body stoichiometry of the consumers (Fig. 3a–b). Hence, the nutritional quality of primary producers increases, improving their suitability as food for omnivorous organisms. Conversely, when C : nutrient ratios of plant material are high, omnivores may overcome this nutrient deficiency by predating on other animals, as was argued for Phryganeidae by Wozniak and Mason (2010). Similar findings were also made in a field study on two *Macrobrachium* shrimp taxa across tropical streams with different dissolved P concentrations (Snyder et al. 2015). The trophic positions of the shrimps, measured by δ^15^N, were lower relative to their potential food sources (i.e., leaf litter, periphyton, and insects) in streams with high dissolved P concentrations, similar to the omnivorous consumers in our study. Since the P content of the shrimps remained homeostatic and there were no differences in P excretion rates, Snyder et al. (2015) suggested that the shrimps shifted their diet from resources with high P contents (e.g., insects) in P‐limited streams to resources low in P (leaf litter) in streams with high dissolved P concentrations.

Nutrient enrichment can also lead to changes in the abundance of prey, providing another explanation for the shifts in trophic position with different productivity levels (Lancaster et al. 2005). For example, Fox et al. (2009) observed a shift in the diet of decapods from feeding mainly as predators in an oligotrophic estuary to feeding mainly as herbivores in a eutrophic estuary, where invertebrate prey were scarce and macroalgae abundant. The ability of aquatic invertebrate omnivores to change their trophic position in response to variation in the abundance and nutritional quality of their potential resources may have important implications for our understanding of species distributions in aquatic ecosystems. In particular, it has been argued that by changing their feeding habits in response to resource availability and/or quality, omnivores are able to exploit a wide range of environments (Lancaster et al. 2005, Wootton 2017).

Our results show changes in the trophic position of aquatic omnivores, but also in the trophic position of the strict carnivore *Notonecta* spp. Backswimmers (*Notonecta* spp.) are fierce predators, feeding on a wide variety of animal prey including zooplankton, mosquito larvae, corixids, and even tadpoles and small fish (Fox 1975, Cronin and Travis 1986, Giller 1986). Hence, the diet of *Notonecta* spp. comprises herbivores, omnivores as well as other carnivorous taxa. Indeed, *Notonecta* spp. occupied the highest trophic position of all investigated taxa at the beginning of the ditch near the nature reserve (TP = 3.9; Fig. 4a), and the observed downward shift in trophic position of this generalist predator along the eutrophication gradient may point at a change in diet from carnivorous toward more herbivorous prey.

Our results relied on the use of the δ^15^N signature of primary producers and consumers to determine the trophic positions of consumer species. Although this method is commonly used (e.g., Post 2002, Middelburg 2014), it faces two major uncertainties. First, the δ^15^N of the primary producers at the baseline can vary both spatially and temporally, e.g., due to agricultural activities (Fig. 3e; see also Boon and Bunn 1994, Cabana and Rasmussen 1996, Peipoch et al. 2012). Motile consumers may integrate δ^15^N over larger spatial and temporal scales than the primary producers (Post 2002). Moreover, the baseline was based on bulk samples of primary producers, whereas some taxa may selectively consume specific plant parts or additional food sources, for example bacteria or detritus (Cross et al. 2005, Peipoch et al. 2012). Therefore, we used multiple species of primary producers and consumers to add robustness to our results, and we sampled all organisms at the end of the growing season to gain a seasonally integrated measure of the stable isotope signature. Second, our approach assumed a fixed ^15^N enrichment of 2.55‰ per trophic level, however, the ^15^N trophic fractionation may typically range between 2–4‰, depending on the studied taxa (Matthews and Mazumder 2008, Middelburg 2014) and the C:N ratio of the consumed food (Adams and Sterner 2000). An important advance of the present study was the use of the same set of taxa along the studied eutrophication gradient, thereby minimizing effects of variation in species composition on trophic fractionation. It seems unlikely that changes in the C:N ratio of the consumed food had a major effect on the ^15^N fractionation per trophic level, as we did not observe a shift in the trophic position of the primary consumers. Species‐specific variation in the baseline and trophic fractionation may have resulted in uncertainty in establishing the precise trophic position of each consumer taxon in our study. Nevertheless, the relative change in trophic position of the different consumer taxa along the eutrophication gradient was evident.

In conclusion, we observed a decrease in the trophic positions of omnivorous aquatic macroinvertebrates and a generalist predator along a eutrophication gradient, presumably due to changes in their diet. For omnivores, this dietary shift is likely to be related to the increased nutritional quality of primary producers in more eutrophic parts of the landscape, which allows for an enhanced consumption of plant material. These concomitant changes in the C:N:P stoichiometry of primary producers and the diet of omnivores will increase grazing pressure on primary producers and enhance the capacity of omnivores to proliferate in ecosystems across a wide range of nutrient conditions. As a consequence, shifts in the diets of aquatic invertebrates induced by increasing eutrophication (due to, e.g., intensification of agriculture and urban expansion) will affect species interactions and food web structure in aquatic ecosystems.

## Supporting information

Appendix S1

Appendix S2

Appendix S3

## Data Availability

Data are available from the Figshare data repository (van der Lee et al. 2020): https://doi.org/10.6084/m9.figshare.13414964.v1.
