# Supplementary material for: Eutrophication induces shifts in the trophic position of invertebrates in aquatic food webs
Source: Ecology. 2021 Feb 13;102(3):e03275. doi: 10.1002/ecy.3275 (PMC7988625; doi:10.1002/ecy.3275)
Supplement: Supplementary file 1 — Appendix S1 [file ECY-102-e03275-s003.pdf]

Supporting Information. van der Lee, G.H., J.A. Vonk, R.C.M. Verdonschot, M.H.S. Kraak, P.F.M. Verdonschot, and J. Huisman. 2021. Eutrophication induces shifts in the trophic position of invertebrates in aquatic food webs. Ecology.

Appendix S1

Table S1. Overview of functional feeding groups assigned according to Merritt & Cummins (1996) and the freshwaterecology.info database (Tachet 2000, Schmidt-Kloiber and Hering 2015). Abbreviations stand for CG = collector-gatherer, SH = shredder, CF = collector-filterer, SC = scraper, PE = predator and PI = piercer.

	Merritt & Cummins (1996)	Freshwaterecology.info						Assigned
		**deposit feeder**	**shredder**	**scraper**	**filter-feeder**	**piercer**	**predator**	
**Primary consumers**								
Amphipoda: Crangonyctidae/Gammaridae	-	0	3	(1)	0	0	0	SH
Asellidae	-	0	3	0	0	0	0	SH
Bithynia spp.	-	0	0	1	2	0	0	CF
Cloeon spp.	CG & SC	3	1	3	0	0	0	CG
Lymnaea spp.	-	0	1	3	0	0	2?	SC
Planorbarius spp	-	0	3	1	0	0	0	SC
Planorbis spp.	-	0	1	2	0	0	0	SC
Sphaeriidae	-	0	0	0	3	0	0	CF
Valvata spp.	-	0	1	2	2	0	0	SC
**Secondary consumers: Potential omnivores**								
Corixinae: *Hesperocorixa* spp./ *Sigara* spp.	PI (carnivore) & CG	0	3	2	0	2	0	PI & CG
Noterus spp.	PR & CG	0	3	0	0	0	3	PR & CG
Phryganeidae: *Agrypnia* spp./*Phryganea* spp.	PR & SH	0	3	1	0	0	1	PR & SH
**Secondary consumers: Strict carnivores**								
Anisoptera	PR	0	0	0	0	0	3	PR
Erpobdella spp.	-	0	0	0	0	0	3	PR
Ilyocoris spp.	PI (carnivore)	0	0	0	0	3	0	PI
Notonecta spp.	PI (carnivore)	0	0	0	0	3	1	PI
Zygoptera	PR	0	0	0	0	0	3	PR

References

Merritt, R. W., & Cummins, K. W. (Eds.). 1996. *An Introduction to the Aquatic Insects of North America*. Kendall Hunt: Dubuque, IA, USA.

Tachet H., Bournaud M., Richoux P., and Usseglio-Polatera P. 2000. Invértebrés d'eau douce: systématique, biologie, écologie. CNRS Editions: Paris.

Schmidt-Kloiber, A., & Hering, D. 2015. www. freshwaterecology. Info – An online tool that unifies, standardises and codifies more than 20,000 European freshwater organisms and their ecological preferences. Ecological Indicators 53: 271-282.
