# Supplementary material for: Eutrophication induces shifts in the trophic position of invertebrates in aquatic food webs
Source: Ecology. 2021 Feb 13;102(3):e03275. doi: 10.1002/ecy.3275 (PMC7988625; doi:10.1002/ecy.3275)
Supplement: Supplementary file 2 — Appendix S2 [file ECY-102-e03275-s002.pdf]

Supporting Information. van der Lee, G.H., J.A. Vonk, R.C.M. Verdonschot, M.H.S. Kraak, P.F.M. Verdonschot, and J. Huisman. 2021. Eutrophication induces shifts in the trophic position of invertebrates in aquatic food webs. Ecology.

Appendix S2

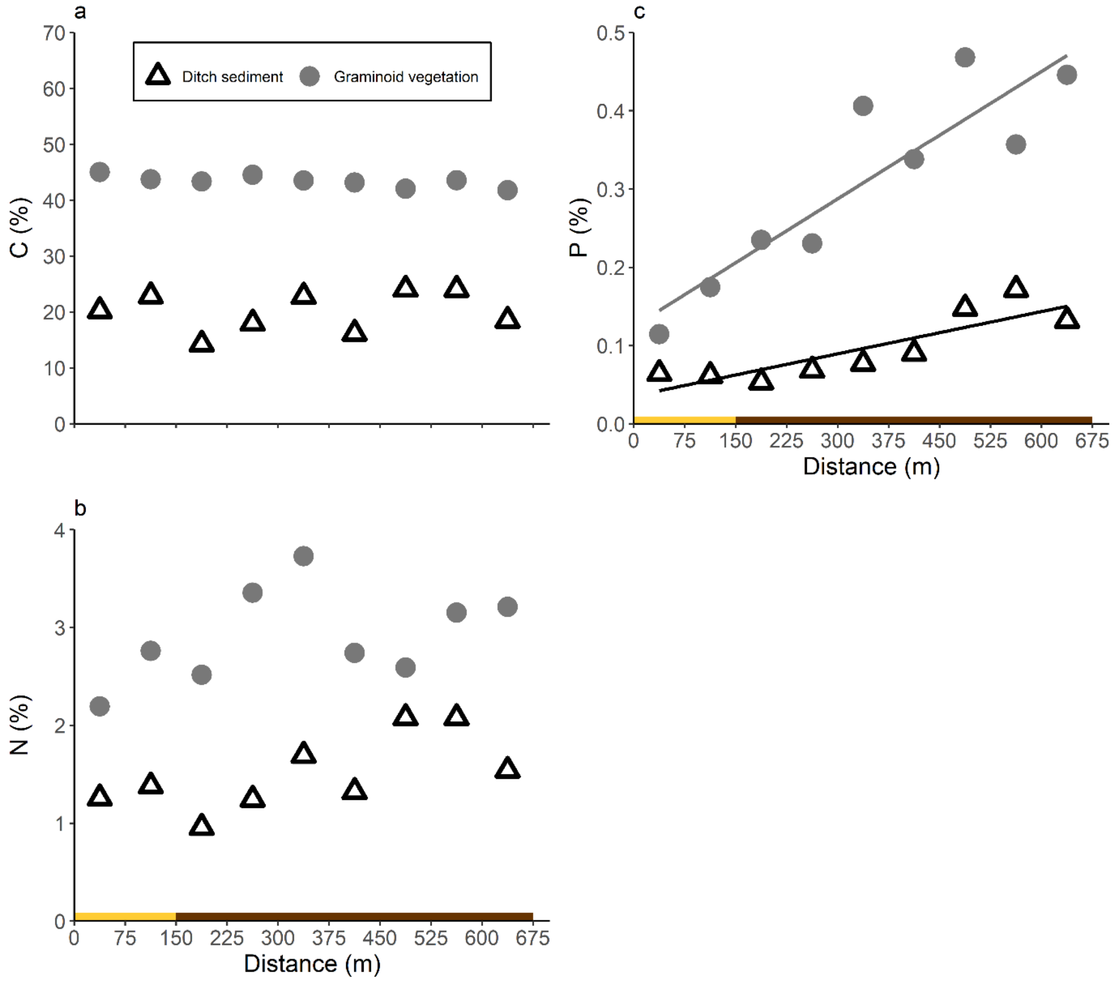

Figure S1. Nutrient content of graminoid vegetation and ditch sediment along the length of the ditch. (a) C content (graminoids: R^2 = 0.59, P = 0.02; sediment: R^2 = 0.03, P = 0.64), (b) N content (graminoids: R^2 = 0.21, P = 0.22; sediment: R^2 = 0.44, P = 0.05), (c) P content (graminoids: R^2 = 0.80, P = 0.001; sediment: R^2 = 0.74, P = 0.003). Lines indicate significant linear regressions (using a Bonferroni corrected significance level of $P < 0.008$; n = 9 in all graphs). The colored bar below the graphs indicates whether the ditch sections were located adjacent to the nature reserve (yellow bar) or agricultural lands (brown bar). Suspended matter was not significant for C content (R^2 = 0.37, P = 0.08), N content (R^2 = 0.06, P = 0.54), and P content (R^2 = 0.08, P = 0.47).
