# Supplementary material for: Eutrophication induces shifts in the trophic position of invertebrates in aquatic food webs
Source: Ecology. 2021 Feb 13;102(3):e03275. doi: 10.1002/ecy.3275 (PMC7988625; doi:10.1002/ecy.3275)
Supplement: Supplementary file 3 — Appendix S3 [file ECY-102-e03275-s001.pdf]

Supporting Information. van der Lee, G.H., J.A. Vonk, R.C.M. Verdonschot, M.H.S. Kraak, P.F.M. Verdonschot, and J. Huisman. 2021. Eutrophication induces shifts in the trophic position of invertebrates in aquatic food webs. Ecology.

Appendix S3

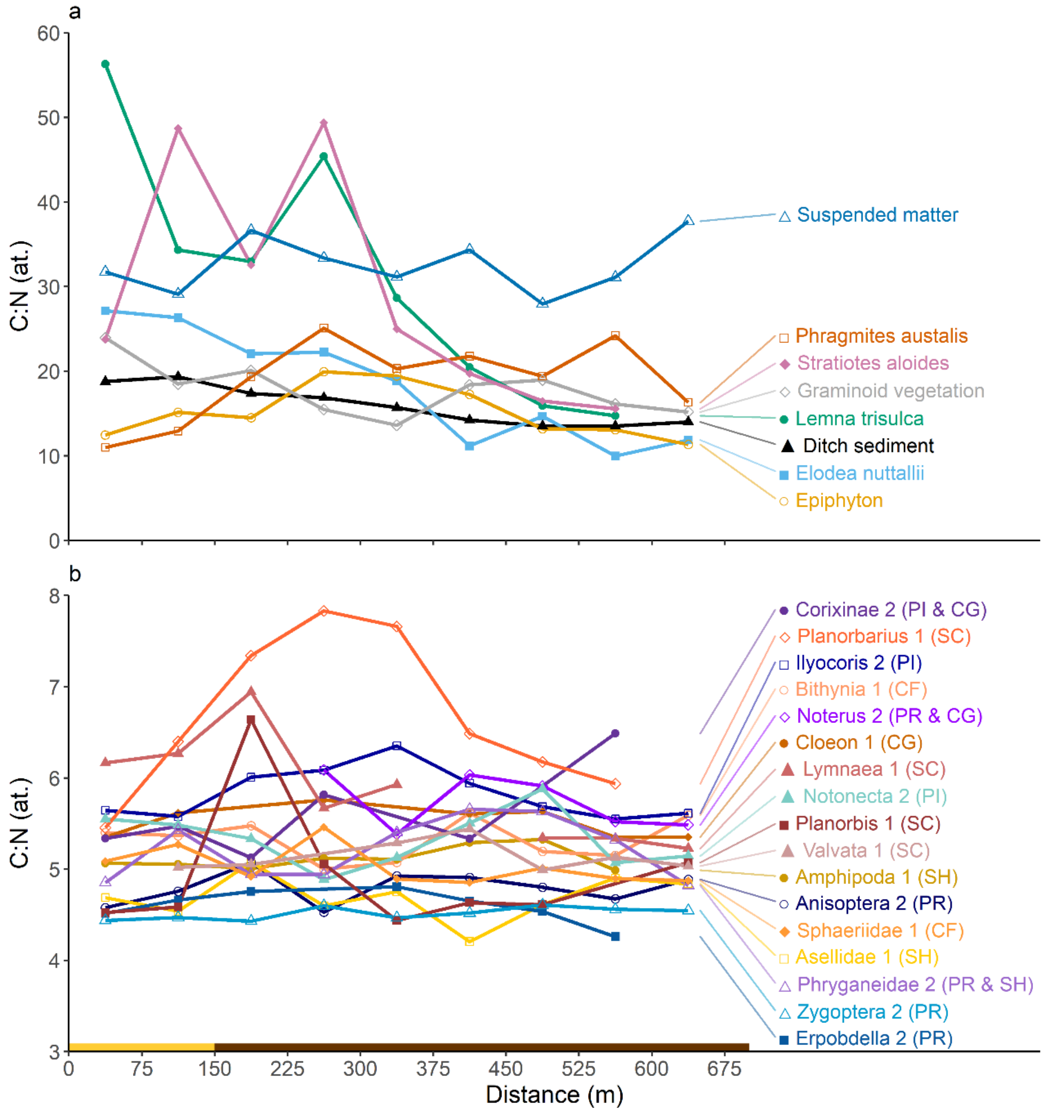

Figure S1. C:N ratios of individual taxa along the length of the ditch. (a) Aquatic primary producers, ditch sediment, suspended matter and graminoid vegetation on the banks. (b) Primary consumers (1) and secondary consumers (2). The abbreviations in brackets stand for functional feeding groups of macroinvertebrates: CF = collector-filterer, CG = collector-gatherer, SC = scraper, SH = shredder, PR = predator and PI = piercer (carnivorous). The colored bar below the graphs indicates whether the ditch sections were located next to the nature reserve (yellow bar) or agricultural lands (brown bar).

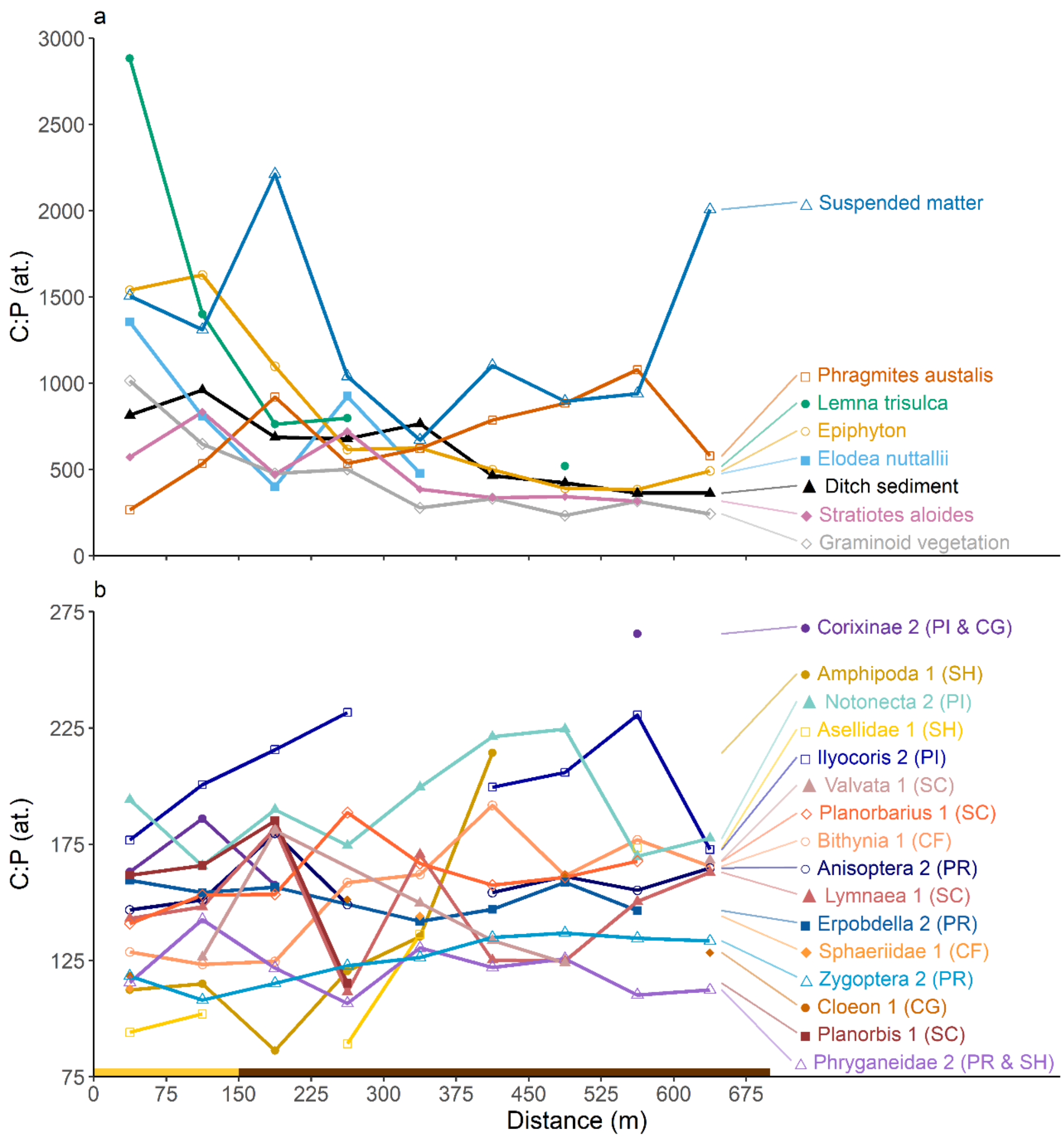

Figure S2. C:P ratios of individual taxa along the length of the ditch. (a) Aquatic primary producers, ditch sediment, suspended matter and graminoid vegetation on the banks. (b) Primary consumers (1) and secondary consumers (2). The abbreviations in brackets stand for functional feeding groups of macroinvertebrates: CF = collector-filterer, CG = collector-gatherer, SC = scraper, SH = shredder, PR = predator and PI = piercer (carnivorous). The colored bar below the graphs indicates whether the ditch sections were located next to the nature reserve (yellow bar) or agricultural lands (brown bar).

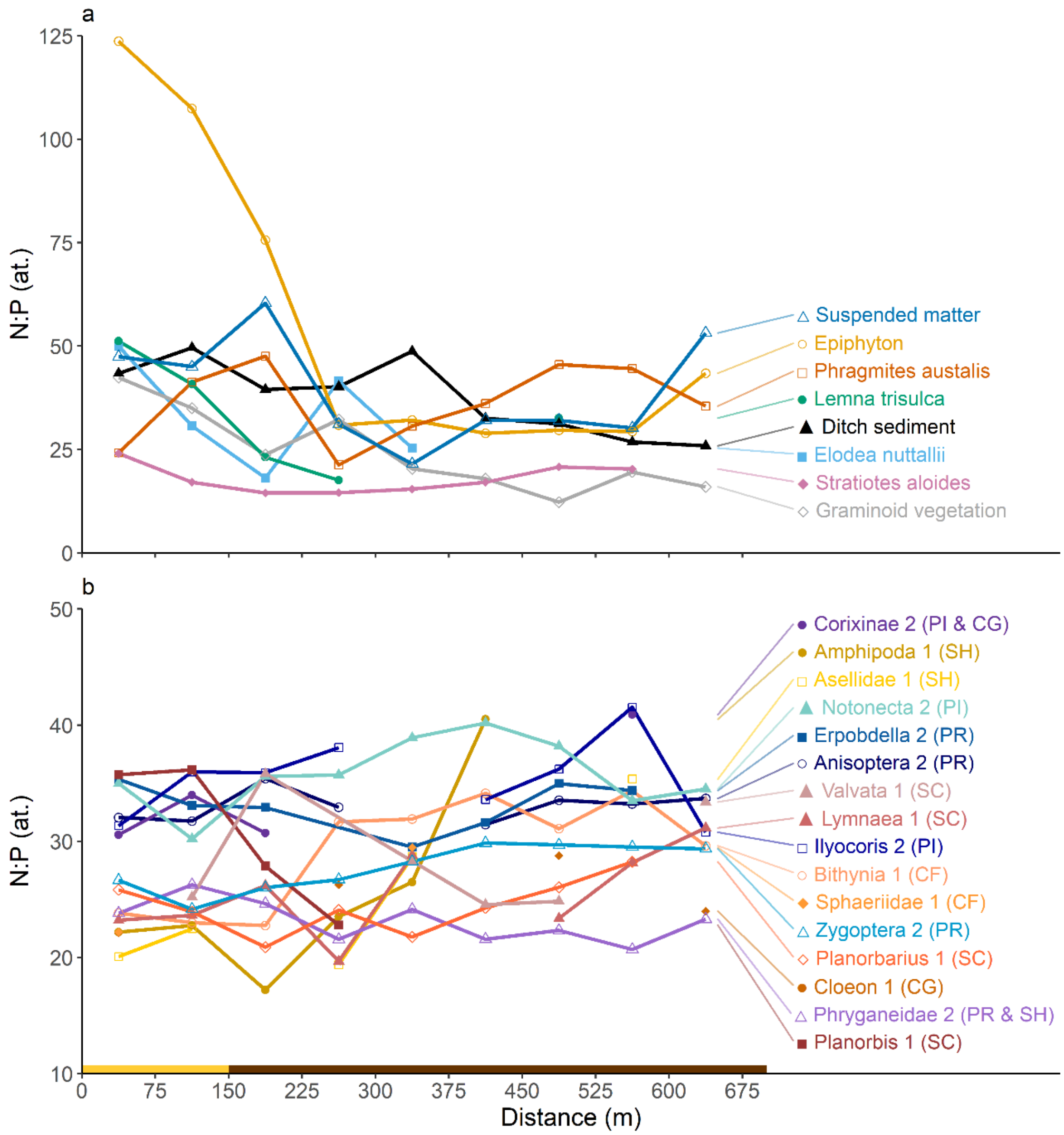

Figure S3. N:P ratios of individual taxa along the length of the ditch. (a) Aquatic primary producers, ditch sediment, suspended matter and graminoid vegetation on the banks. (b) Primary consumers (1) and secondary consumers (2). The abbreviations in brackets stand for functional feeding groups of macroinvertebrates: CF = collector-filterer, CG = collector-gatherer, SC = scraper, SH = shredder, PR = predator and PI = piercer (carnivorous). The colored bar below the graphs indicates whether the ditch sections were located next to the nature reserve (yellow bar) or agricultural lands (brown bar).

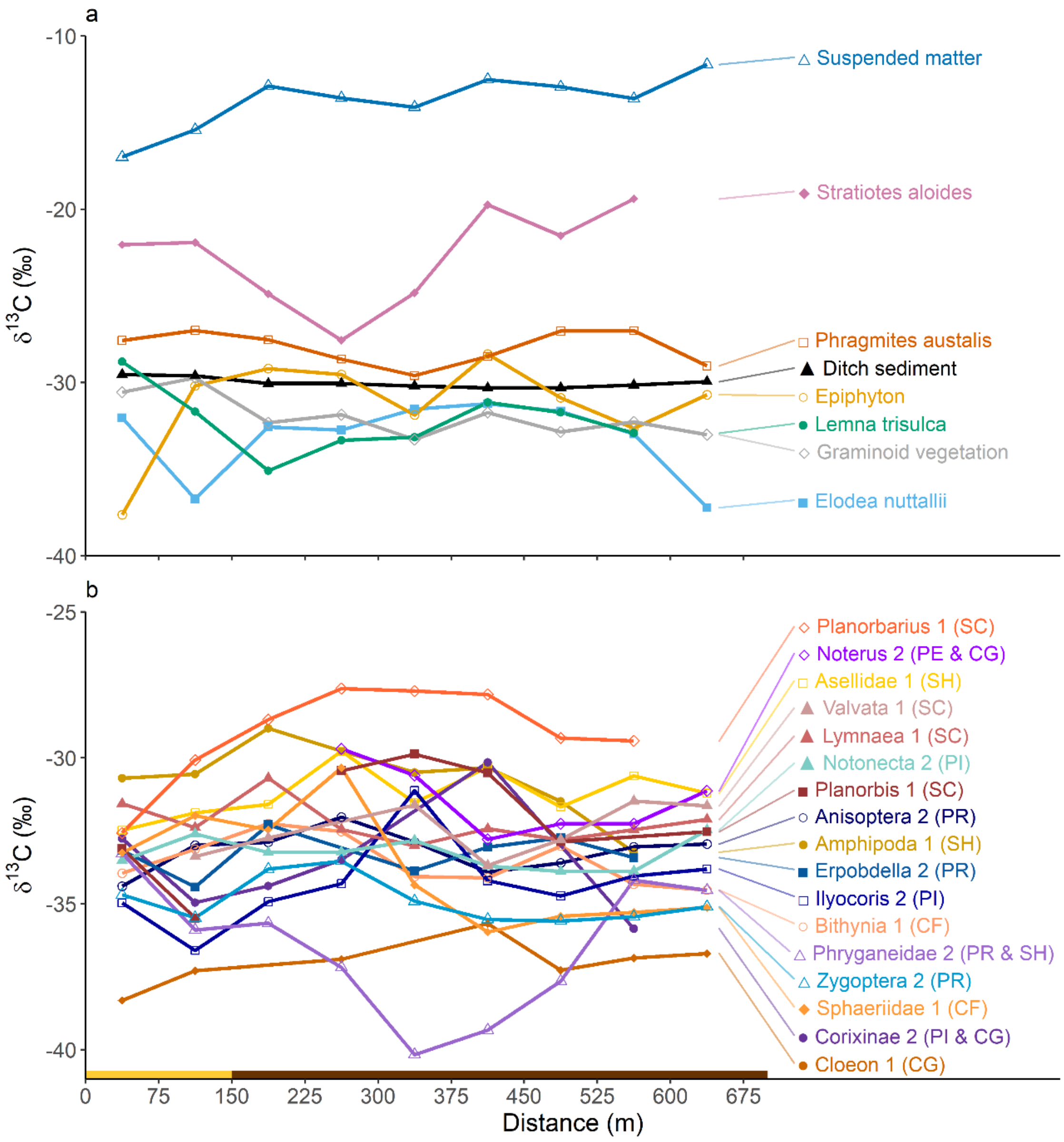

Figure S4. Carbon isotope signature ($\delta^{13}C$) of individual taxa along the length of the ditch. (a) Aquatic primary producers, ditch sediment, suspended matter and graminoid vegetation on the banks. (b) Primary consumers (1) and secondary consumers (2). The abbreviations in brackets stand for functional feeding groups of macroinvertebrates: CF = collector-filterer, CG = collector-gatherer, SC = scraper, SH = shredder, PR = predator and PI = piercer (carnivorous). The colored bar below the graphs indicates whether the ditch sections were located next to the nature reserve (yellow bar) or agricultural lands (brown bar).

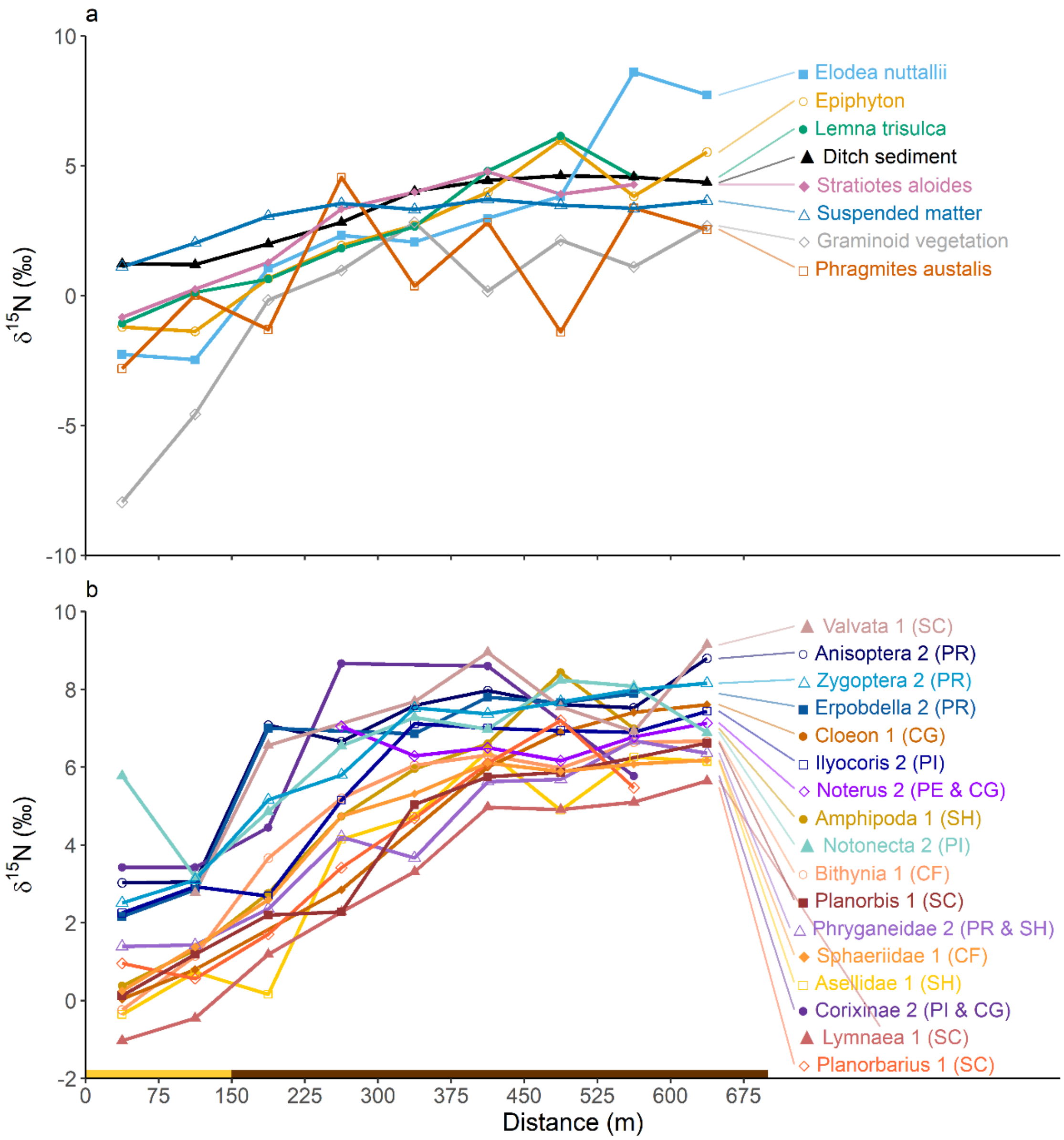

Figure S5. Nitrogen isotope signature ($\delta^{15}N$) of individual taxa along the length of the ditch. (a) Aquatic primary producers, ditch sediment, suspended matter and graminoid vegetation on the banks. (b) Primary consumers (1) and secondary consumers (2). The abbreviations in brackets stand for functional feeding groups of macroinvertebrates: CF = collector-filterer, CG = collector-gatherer, SC = scraper, SH = shredder, PR = predator and PI = piercer (carnivorous). The colored bar below the graphs indicates whether the ditch sections were located next to the nature reserve (yellow bar) or agricultural lands (brown bar).

Table S1. Elemental stoichiometry of environmental parameters, primary producers, and consumers. Reported are the mean and standard deviation (SD) over the length of the ditch, and the R^2 and P values resulting from linear regressions of the indicated stoichiometric ratios as function of the distance along the ditch. Significant regressions are presented in bold (Bonferroni corrected, $P < 0.0083$). For individual taxa the linear regression was performed only if n > 4. n/a = not applicable.

		C:N (at.)					C:P (at.)					N:P (at.)				
		Mean	SD	R^2	*P*	n	Mean	SD	R^2	*P*	n	Mean	SD	R^2	*P*	n
Environment	Graminoid vegetation	17.8	3.1	0.37	0.08	9	448	254	**0.73**	**0.003**	9	24.4	10.0	**0.75**	**0.003**	9
	Ditch sediment	15.9	2.3	**0.90**	**<0.001**	9	612	218	**0.83**	**0.001**	9	37.5	8.9	**0.70**	**0.005**	9
	Suspended matter	32.6	3.3	0.04	0.62	9	1298	521	0.02	0.71	9	39.2	12.8	0.08	0.47	9
Primary producers	*Elodea nuttallii*	18.3	6.6	**0.89**	**<0.001**	9	792	384	0.46	0.21	5	33.1	12.7	0.23	0.42	5
	Epiphyton	15.1	3.1	0.06	0.54	9	808	489	**0.78**	**0.002**	9	55.7	37.3	0.63	0.01	9
	Lemna trisulca	31.1	14.5	**0.77**	**0.004**	8	1272	957	0.60	0.12	5	33.1	13.5	0.24	0.40	5
	Phragmites australis	18.9	4.8	0.25	0.17	9	689	249	0.32	0.12	9	36.3	9.4	0.10	0.40	9
	Stratiotes aloides	28.9	13.5	0.37	0.11	8	497	194	0.56	0.03	8	18.0	3.4	0.00	0.99	8
Primary consumers	Amphipoda	5.1	0.1	0.15	0.34	8	131	44	0.54	0.10	6	25.4	8.0	0.54	0.10	6
	Asellidae	4.7	0.2	0.00	0.86	9	119	36	0.78	0.05	5	25.2	6.8	0.77	0.05	5
	Bithynia spp.	5.3	0.2	0.00	0.93	9	155	24	0.62	0.01	9	29.2	4.7	0.55	0.02	9
	Cloeon spp.	5.5	0.2	0.06	0.61	7	140	20	n/a	n/a	4	25.3	2.9	n/a	n/a	4
	Lymnaea spp.	5.9	0.6	0.64	0.02	8	147	23	0.00	0.94	9	25.5	3.7	0.36	0.12	8
	Planorbarius spp.	6.7	0.9	0.00	0.97	8	161	14	0.20	0.26	8	24.4	2.4	0.19	0.28	8
	Planorbis spp.	4.9	0.7	0.01	0.83	8	157	30	n/a	n/a	4	30.6	6.5	n/a	n/a	4
	Sphaeriidae	5.0	0.2	0.27	0.15	9	128	22	n/a	n/a	2	25.8	5.1	n/a	n/a	2
	Valvata spp.	5.1	0.2	0.00	0.93	7	147	23	0.00	0.91	6	28.7	4.8	0.00	0.92	6
Secondary consumers	Corixinae	5.6	0.5	0.35	0.21	6	193	50	n/a	n/a	4	34.0	4.8	n/a	n/a	4
	Noterus spp.	5.7	0.3	0.22	0.35	6	n/a	n/a	n/a	n/a	0	n/a	n/a	n/a	n/a	0
	Phryganeidae	5.2	0.3	0.05	0.56	9	121	11	0.12	0.35	9	23.1	1.8	0.36	0.09	9
	Anisoptera	4.8	0.2	0.04	0.59	9	158	11	0.06	0.57	8	33.0	1.3	0.05	0.60	8
	Erpobdella spp.	4.6	0.2	0.19	0.33	7	152	7	0.25	0.26	7	33.1	2.1	0.01	0.88	7
	Ilyocoris spp.	5.8	0.3	0.03	0.68	9	204	22	0.00	0.91	8	35.4	3.5	0.02	0.73	8
	Notonecta spp.	5.3	0.3	0.03	0.66	9	191	21	0.02	0.73	9	35.8	3.0	0.07	0.49	9
	Zygoptera	4.5	0.1	0.44	0.05	9	126	10	**0.76**	**0.002**	9	27.8	2.0	**0.72**	**0.004**	9

Table S2. Stable isotope signature of environmental parameters, primary producers, primary consumers, and consumers. Reported are the mean and standard deviation (SD) over the length of the ditch, and the R^2 and P values resulting from linear regression of trophic position as function of the distance along the ditch. Significant regressions are indicated in bold (Bonferroni corrected, $P < 0.0083$). For individual taxa the linear regression was performed only if n > 4. n/a = not applicable.

		$\delta^{13}C$ (‰)					$\delta^{15}N$ (‰)					Trophic position				
		Mean	SD	R^2	*P*	n	Mean	SD	R^2	*P*	n	Mean	SD	R^2	*P*	n
Environment	Graminoid vegetation	-32.0	1.2	0.51	0.03	9	-0.3	3.6	0.64	0.01	9	n/a	n/a	n/a	n/a	n/a
	Ditch sediment	-30.0	0.3	0.43	0.06	9	3.2	1.5	**0.86**	**<0.001**	9	n/a	n/a	n/a	n/a	n/a
	Suspended matter	-13.7	1.6	0.62	0.01	9	3.0	0.9	0.62	0.01	9	n/a	n/a	n/a	n/a	n/a
Primary producers	*Elodea nuttallii*	-33.2	2.2	0.02	0.75	9	2.7	3.8	**0.90**	**<0.001**	9	n/a	n/a	n/a	n/a	n/a
	Epiphyton	-31.2	2.7	0.09	0.43	9	2.4	2.7	**0.88**	**<0.001**	9	n/a	n/a	n/a	n/a	n/a
	Lemna trisulca	-32.2	1.9	0.07	0.52	8	2.5	2.5	**0.91**	**<0.001**	8	n/a	n/a	n/a	n/a	n/a
	Phragmites australis	-28.0	1.0	0.05	0.56	9	0.9	2.5	0.29	0.14	9	n/a	n/a	n/a	n/a	n/a
	Stratiotes aloides	-22.7	2.8	0.16	0.32	8	2.6	2.1	**0.82**	**0.002**	8	n/a	n/a	n/a	n/a	n/a
Primary consumers	Amphipoda	-30.7	1.3	0.40	0.09	8	4.6	2.9	**0.91**	**<0.001**	8	2.0	0.4	0.24	0.22	8
	Asellidae	-31.2	0.8	0.19	0.24	9	3.7	2.7	**0.82**	**0.001**	9	1.5	0.3	0.02	0.74	9
	Bithynia spp.	-33.5	0.8	0.25	0.17	9	4.6	2.5	**0.80**	**0.001**	9	1.9	0.3	0.02	0.75	9
	Cloeon spp.	-37.0	0.8	0.34	0.17	7	4.5	3.2	**0.97**	**<0.001**	7	1.7	0.4	0.32	0.18	7
	Lymnaea spp.	-32.2	0.7	0.18	0.25	9	2.9	2.5	**0.94**	**<0.001**	9	1.2	0.2	0.02	0.75	9
	Planorbarius spp.	-29.2	1.6	0.25	0.21	8	3.8	2.5	**0.86**	**0.001**	8	1.7	0.4	0.00	0.92	8
	Planorbis spp.	-31.2	3.1	0.00	0.97	8	3.6	2.5	**0.92**	**<0.001**	8	1.6	0.4	0.00	0.91	8
	Sphaeriidae	-33.8	1.9	0.48	0.04	9	4.3	2.3	**0.84**	**0.001**	9	1.8	0.2	0.22	0.20	9
	Valvata spp.	-32.5	0.9	0.31	0.20	7	7.1	2.1	0.56	0.05	7	2.7	0.5	0.20	0.31	7
Secondary consumers	Corixinae	-33.6	2.0	0.00	0.94	6	5.7	2.4	0.35	0.21	6	2.6	0.7	0.41	0.17	6
	Noterus spp.	-31.5	1.2	0.27	0.29	6	6.7	0.4	0.04	0.69	6	2.1	0.4	**0.91**	**0.003**	6
	Phryganeidae	-36.4	2.3	0.01	0.76	9	4.2	2.1	**0.93**	**<0.001**	9	1.7	0.2	0.45	0.05	9
	Anisoptera	-33.2	0.7	0.02	0.69	9	6.6	2.1	**0.72**	**0.004**	9	2.7	0.5	0.27	0.15	9
	Erpobdella spp.	-33.3	0.7	0.03	0.72	7	6.0	2.4	0.76	0.01	7	2.6	0.4	0.10	0.50	7
	Ilyocoris spp.	-34.3	1.4	0.16	0.28	9	5.4	2.2	**0.82**	**0.001**	9	2.2	0.4	0.19	0.24	9
	Notonecta spp.	-33.3	0.5	0.01	0.76	9	6.4	1.6	0.56	0.02	9	2.6	0.6	0.55	0.02	9
	Zygoptera	-34.9	0.8	0.18	0.26	9	6.1	2.1	**0.87**	**<0.001**	9	2.5	0.3	0.23	0.20	9
